# Years of Potential Life Lost and Mean Age of Adults Experiencing Nontraumatic, Out-of-Hospital Cardiac Arrests — Chicago, 2014–2021

**DOI:** 10.15585/mmwr.mm7309a2

**Published:** 2024-03-07

**Authors:** Shaveta Khosla, Marina Del Rios, Pavitra Kotini-Shah, Joseph Weber, Terry Vanden Hoek, Illinois Heart Rescue

**Affiliations:** ^1^Department of Emergency Medicine, University of Illinois Chicago, Chicago, Illinois; ^2^Department of Emergency Medicine, University of Iowa, Carver College of Medicine, Iowa City, Iowa; ^3^Department of Emergency Medicine, John H. Stroger, Jr. Hospital, Chicago, Illinois.

**Summary**

**What is already known about this topic?**

Approximately 1,000 out-of-hospital cardiac arrests are assessed by emergency medical services in the United States every day, and approximately 90% of patients do not survive.

**What is added by this report?**

The overall years of potential life lost increased from 52,044 years during 2014–2015 to 88,788 years during 2020–2021, and the mean age of out-of-hospital cardiac arrests in Chicago decreased progressively from 64.7 years during 2014–2015, to 62.7 years during 2020–2021.

**What are the implications for public health practice?**

Increased public awareness of the risk for cardiac arrest and knowledge of how to intervene as a bystander could help decrease associated mortality. Improved understanding of the reasons for the observed decrease in mean age at cardiac arrest could help guide prevention efforts.

## Abstract

Approximately 1,000 out-of-hospital cardiac arrests (OHCAs) are assessed by emergency medical services in the United States every day, and approximately 90% of patients do not survive, leading to substantial years of potential life lost (YPLL). Chicago emergency medical services data were used to assess changes in mean age and YPLL from nontraumatic OHCA in adults in biennial cycles during 2014–2021. Among 21,070 reported nontraumatic OHCAs during 2014–2021, approximately 60% occurred among men and 57% among non-Hispanic Black or African American (Black) persons. YPLL increased from 52,044 during 2014–2015 to 88,788 during 2020–2021 (p = 0.002) and mean age decreased from 64.7 years during 2014–2015, to 62.7 years during 2020–2021. Decrease in mean age occurred among both men (p<0.001) and women (p = 0.002) and was largest among Black men. Mean age decreased among patients without presumed cardiac etiology from 56.3 to 52.5 years (p<0.001) and among patients with nonshockable rhythm from 65.5 to 62.7 years (p<0.001). Further study is needed to assess whether similar trends are occurring elsewhere, and to understand the mechanisms that underlie these trends in Chicago because these mechanisms could help guide prevention efforts. Increased public awareness of the risk of cardiac arrest and knowledge of how to intervene as a bystander could help decrease associated mortality.

## Introduction

Approximately 1,000 out-of-hospital cardiac arrests (OHCAs) are assessed by emergency medical services (EMS) in the United States every day. Approximately 90% result in death* (*1*,*2*), leading to substantial years of potential life lost (YPLL). YPLL due to OHCA are higher than that from other causes of death (*3*). Recent decreases in mean age of in-hospital cardiac arrest patients have been reported (*4*,*5*); however, whether such a decrease has occurred among OHCA patients is not known. This study describes changes in YPLL from OHCA and mean age at OHCA among nontraumatic cases in adults in Chicago during 2014–2021.

## Methods

During 2014–2021, a total of 22,158 OHCAs were reported to Chicago’s Cardiac Arrest Registry to Enhance Survival (CARES) and served by Chicago EMS. The following cases were excluded: pediatric cases (among persons aged <18 years; 588), trauma cases (462), and cases missing patient age (38), resulting in 21,070 cases included. Annual data were combined to create 2-year cycles. Mean values were calculated for continuous variables. Frequencies were calculated for categorical variables.

For YPLL calculations, patients who were missing survival information (35) or who survived to hospital discharge (1,756) were excluded. YPLL was calculated using a standard age of 75 years^†^ (i.e., among patients younger than age 75 years who died, age was subtracted from 75 and then summed). Patients aged ≥75 years (5,541) contributed zero YPLL. Deaths that occurred before or during hospital admission (13,738) contributed to positive YPLL. For the YPLL rate, the denominator was the adult U.S. Census Bureau population estimate (*6*) of the first year in each 2-year cycle. Rates were expressed per 100,000 adult population per biennial cycle. Trends in mean age were calculated using linear regression models; p-value of the slope (i.e., p-value corresponding to the t-test for whether slope is significantly different from zero) was reported. This study was determined to be not human subjects research by the Institutional Review Board at University of Illinois Chicago.^§^

If the first monitored rhythm was categorized as ventricular fibrillation, unknown shockable rhythm, or ventricular tachycardia, the rhythm was considered shockable. If the first monitored rhythm was categorized as asystole, idioventricular or pulseless electrical activity, or unknown unshockable rhythm, the rhythm was considered not shockable. Cardiac etiology was presumed unless the arrest was known or likely to have had a noncardiac cause (e.g., drowning, asphyxia, electrocution, overdose, poisoning, or hemorrhage). More details on the variables can be found in CARES data dictionary (*7*). SAS software (version 9.4; SAS Institute) was used for statistical analysis.

## Results

**FIGURE 1:**
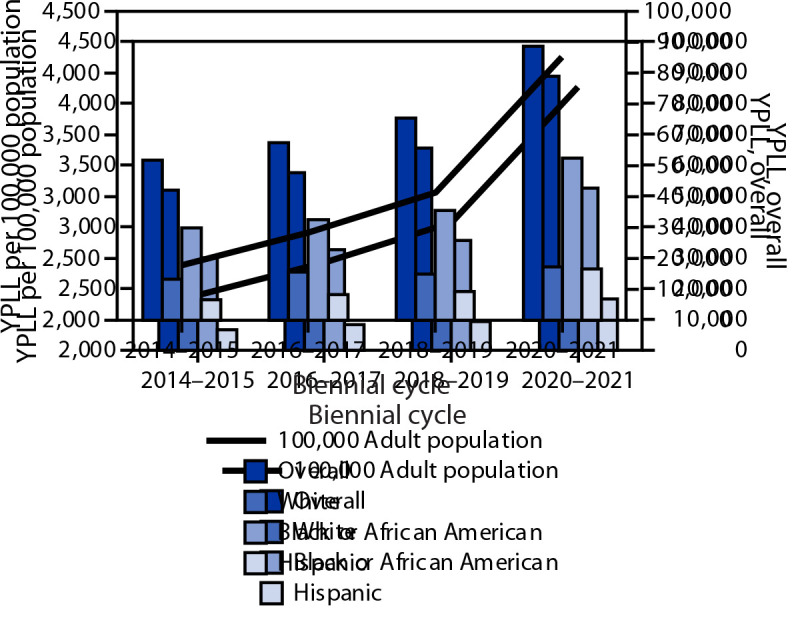
Years of potential life lost from nontraumatic out-of-hospital cardiac arrest among adults per 100,000 population,* overall, and by race and ethnicity^†^ — Chicago, 2014–2021 **Abbreviation:** YPLL = years of potential life lost. * YPLL rate was calculated using the adult U.S. Census Bureau population estimate of the baseline year as the denominator (i.e., 2014 population estimate for the 2014–2015 cycle, 2016 population estimate for the 2016–2017 cycle, and so on). ^†^ Persons of Hispanic or Latino (Hispanic) origin might be of any race but are categorized as Hispanic; all racial groups are non-Hispanic. Asian group was not presented separately because of a smaller number of cases that were applicable to YPLL calculations, and a resulting higher variability in YPLL.

Approximately 60% of the 21,070 adult OHCAs occurred among men, 57% among Black or African American (Black) adults, 26% among White adults, 12% among Hispanic or Latino (Hispanic) adults, and 2% among Asian adults; the rest were classified as other.^¶^ The percentage of OHCAs increased over time among adults aged 26–45 and 56–65 years, and decreased among those aged >75 years. Consistent with this pattern, overall YPLL increased from 52,044 during 2014–2015 to 88,788 during 2020–2021 (p = 0.002). YPLL among Black adults increased from 29,956 during 2014–2015 to 52,477 during 2020–2021 (p = 0.003) (Figure 1). YPLL per 100,000 adult population per biennial cycle increased from 2,450 during 2014–2015 to 4,136 during 2020–2021.

**FIGURE 2:**
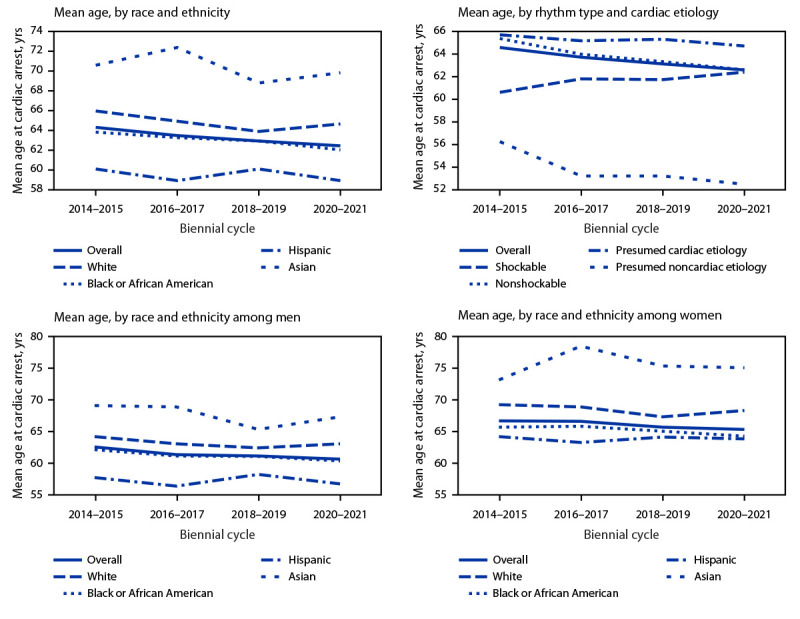
Trends in mean age for out-of-hospital cardiac arrest among adults, by various characteristics* — Chicago, 2014–2021 * Persons of Hispanic or Latino (Hispanic) origin might be of any race but are categorized as Hispanic; all racial groups are non-Hispanic.

The mean age for the entire study period, 63.5 years (Table), decreased from 64.7 during 2014–2015 to 62.7 during 2020–2021 (p<0.001). The mean age at which OHCA occurred among men decreased from 62.5 to 60.6 years, with a biennial change of −0.6 years (p<0.001); among women, the mean age decreased from 67.6 to 66.1 years with a biennial change of −0.5 years (p = 0.002). The downward trend began before the COVID-19 pandemic (2014–2019). Among Black adults, the mean age decreased from 64.2 years during 2014–2015 to 62.3 years during 2020–2021 (p<0.001) and among White adults decreased from 66.5 to 65.1 years (p = 0.02). Mean age was consistently lowest among Hispanic adults and highest among Asian adults (Figure 2). When race and ethnicity and sex are considered together, the largest decrease in mean age occurred among Black men (from 62.1 years during 2014–2015 to 60.3 years during 2020–2021; biennial change of −0.6 years; p<0.001).

**TABLE:** Characteristics of adult nontraumatic out-of-hospital cardiac arrests, by biennial cycles — Chicago, 2014–2021

Characteristic	No. (%)				
	Overall N = 21,070	2014–2015 n = 4,486	2016–2017 n = 4,700	2018–2019 n = 5,233	2020–2021 n = 6,651
**Mean age, yrs**	**63.5**	64.7	63.8	63.2	62.7
**Age group, yrs**					
18–25	**414 (2.0)**	82 (1.8)	102 (2.2)	83 (1.6)	147 (2.2)
26–35	**986 (4.7)**	164 (3.7)	222 (4.7)	249 (4.8)	351 (5.3)
36–45	**1,664 (7.9)**	326 (7.3)	337 (7.2)	432 (8.3)	569 (8.6)
46–55	**3,401 (16.1)**	727 (16.2)	771 (16.4)	856 (16.4)	1,047 (15.7)
56–65	**4,949 (23.5)**	1,015 (22.6)	1,098 (23.4)	1,249 (23.9)	1,587 (23.9)
66–75	**4,181 (19.8)**	874 (19.5)	904 (19.2)	1,069 (20.4)	1,334 (20.1)
76–85	**3,348 (15.9)**	784 (17.5)	778 (16.6)	805 (15.4)	981 (14.8)
>85	**2,127 (10.1)**	514 (11.5)	488 (10.4)	490 (9.4)	635 (9.6)
Race and ethnicity*					
Asian	**513 (2.4)**	120 (2.7)	84 (1.8)	139 (2.7)	170 (2.6)
Black or African American	**11,932 (56.6)**	2,516 (56.1)	2,663 (56.7)	2,868 (54.8)	3,885 (58.4)
White	**5,522 (26.2)**	1,320 (29.4)	1,399 (29.8)	1,289 (24.6)	1,514 (22.8)
Hispanic or Latino	**2,606 (12.4)**	444 (9.9)	525 (11.2)	625 (11.9)	1,012 (15.2)
Other	**497 (2.4)**	86 (1.9)	29 (0.6)	312 (6.0)	70 (1.0)
**Sex**					
Men	**12,683 (60.2)**	2,590 (57.7)	2,813 (59.8)	3,172 (60.6)	4,108 (61.8)
Women	**8,386 (39.8)**	1,896 (42.3)	1,887 (40.2)	2,061 (39.4)	2,542 (38.2)
**Shockable rhythm^†^**					
Yes	**2,880 (13.7)**	772 (17.2)	733 (15.6)	692 (13.2)	683 (10.3)
No	**18,190 (86.3)**	3,714 (82.8)	3,967 (84.4)	4,541 (86.8)	5,968 (89.7)
**Presumed cardiac etiology^§^**					
Yes	**17,854 (84.7)**	3,953 (88.1)	4,116 (87.6)	4,279 (81.8)	5,506 (82.8)
No	**3,216 (15.3)**	533 (11.9)	584 (12.4)	954 (18.2)	1,145 (17.2)

* Persons of Hispanic or Latino (Hispanic) origin might be of any race but are categorized as Hispanic; all racial groups are non-Hispanic. “Other” includes those with mixed, unknown, or other race and ethnicity.

^†^ If the first monitored rhythm was categorized as ventricular fibrillation, unknown shockable rhythm, or ventricular tachycardia, the rhythm was considered shockable. If the first monitored rhythm was categorized as asystole, idioventricular/pulseless electrical activity, or unknown unshockable rhythm, the rhythm was considered not shockable.

^§^ Cardiac etiology was presumed unless the arrest was known or likely to have had a noncardiac cause (e.g., drowning, asphyxia, electrocution, overdose, poisoning, or hemorrhage).

Approximately 14% of OHCAs had an initial shockable rhythm, and 84.7% had a presumed cardiac etiology (Table). The mean age of persons without presumed cardiac etiology decreased from 56.3 years during 2014–2015 to 52.5 years during 2020–2021 (biennial change = −1.0 years; p<0.001) (Figure 2). The mean age of patients presumed to have cardiac etiology decreased from 65.8 years during 2014–2015 to 64.8 during 2020–2021 (biennial change = −0.3 years; p = 0.007). The mean age of patients with nonshockable rhythm decreased from 65.5 years during 2014–2015 to 62.7 years during 2020–2021 (biennial change = −0.9 years; p<0.001). Cases with shockable rhythm did not show this decrease in mean age; instead, an increase in mean age occurred (p = 0.03). 

## Discussion

The mean age of OHCA in Chicago decreased from 2014–2015 to 2020–2021 overall, for men and women, Black and White adults, as well as for cases in persons with or without presumed cardiac etiology and for nonshockable rhythm type. Decreases in mean age were more pronounced for patients without presumed cardiac etiology, those with nonshockable rhythm, men, and Black adults. Survival for OHCA is low (*1*–*3*), and earlier age of death results in a larger number of YPLL.

The decrease in mean age at OHCA occurrence among patients with noncardiac etiology might be related to the increase in opioid-related overdose (*8*), which coincides with the steady increase in nonshockable cases over time with a substantial decrease in mean age. Although this change could be related to overdose, the pandemic might have played a role during 2020–2021. It is not fully known why shockable cases did not reflect this trend of decreasing mean age. The larger decrease in mean age of persons experiencing cardiac arrest among men and among Black adults increased disparities that already existed on the basis of race and sex.

### Limitations

The findings in this report are subject to at least three limitations. First, because of the limited number of cases, racial and ethnic groups other than Asian, Black, White, and Hispanic could not be assessed individually. Second, cases that occurred near the city boundary of Chicago might have been served by either Chicago EMS or a different EMS agency. Finally, the contribution of specific causes of OHCA, such as drug overdose or thromboembolic events associated with COVID-19, to the observed trends could not be assessed in this analysis.

### Implications for Public Health Practice

This analysis shows a concerning trend at the population level that cannot be entirely attributed to the COVID-19 pandemic because it began before the pandemic. Additional research and enhanced surveillance mechanisms (e.g., hotspot identification and cross-linkage of socioeconomic, comorbidity, substance use, and medication use data) could help elucidate the factors contributing to these observed trends and guide prevention efforts (*9*,*10*). Promotion of regular health checks is important to identify persons at risk for OHCA and intervene appropriately. Efforts to increase public awareness of the risk of cardiac arrest and knowledge of how to intervene as a bystander could help decrease mortality associated with OHCA. Improved understanding of the mechanisms that underlie the trends observed in Chicago could help guide prevention efforts. Similar analyses in other jurisdictions could help determine whether trends observed in Chicago are more widespread.

## Footnotes

*
https://cpr.heart.org/en/resources/cpr-facts-and-stats

† Many departments of health in U.S. states use age 75 years as the benchmark for YPLL calculations. https://health.mo.gov/data/ypll/; https://www-doh.state.nj.us/doh-shad/view/sharedstatic/YearsOfPotentialLifeLost.pdf

§ 45 C.F.R. part 46.101(c); 21 C.F.R. part 56.

¶ Persons of Hispanic or Latino (Hispanic) origin might be of any race but are categorized as Hispanic; all racial groups are non-Hispanic.
